# Chromatin Dynamics in Digestive System Cancer: Commander and Regulator

**DOI:** 10.3389/fonc.2022.935877

**Published:** 2022-07-29

**Authors:** Zeru Li, Bangbo Zhao, Cheng Qin, Yuanyang Wang, Tianhao Li, Weibin Wang

**Affiliations:** Department of General Surgery, State Key Laboratory of Complex Severe and Rare Diseases, Peking Union Medical College Hospital, Chinese Academy of Medical Sciences and Peking Union Medical College, Beijing, China

**Keywords:** digestive system tumor, epigenetics, chromatin dynamics, targeted therapy, clinical trials

## Abstract

Digestive system tumors have a poor prognosis due to complex anatomy, insidious onset, challenges in early diagnosis, and chemoresistance. Epidemiological statistics has verified that digestive system tumors rank first in tumor-related death. Although a great number of studies are devoted to the molecular biological mechanism, early diagnostic markers, and application of new targeted drugs in digestive system tumors, the therapeutic effect is still not satisfactory. Epigenomic alterations including histone modification and chromatin remodeling are present in human cancers and are now known to cooperate with genetic changes to drive the cancer phenotype. Chromatin is the carrier of genetic information and consists of DNA, histones, non-histone proteins, and a small amount of RNA. Chromatin and nucleosomes control the stability of the eukaryotic genome and regulate DNA processes such as transcription, replication, and repair. The dynamic structure of chromatin plays a key role in this regulatory function. Structural fluctuations expose internal DNA and thus provide access to the nuclear machinery. The dynamic changes are affected by various complexes and epigenetic modifications. Variation of chromatin dynamics produces early and superior regulation of the expression of related genes and downstream pathways, thereby controlling tumor development. Intervention at the chromatin level can change the process of cancer earlier and is a feasible option for future tumor diagnosis and treatment. In this review, we introduced chromatin dynamics including chromatin remodeling, histone modifications, and chromatin accessibility, and current research on chromatin regulation in digestive system tumors was also summarized.

## Introduction

Following a 19-year effort to sequence the full human genome, the landscape of human cancers began to be revealed. One of the most valuable results of this genome sequencing effort was that epigenetic and chromatin remodeling-centered processes were closely linked to cancer development (1). Cancer occurrence and progression are consequences of disruption of the mechanisms that regulate critical progress, such as cell proliferation, metabolism, apoptosis, and invasion, as well as other hallmark biological behaviors in cancer (1, 2). These disruptions are known as commonly caused by early alterations at the chromatin and DNA levels. Chromatin is a multidimensional complex structure of genetic material that existed in the nucleus of interphase cells consisting of DNA, histones, non-histones, and a small amount of RNA. Genetic material evolves from DNA to densely packed chromosomes through four main stages, namely, the primary structure (nucleosomes), the secondary structure (solenoids), the tertiary structure (supersolenoid), and the quaternary chromatin (chromosome) (2). Chromatin remodeling and chromatin accessibility are important concepts of epigenetics. Chromatin remodeling is a molecular mechanism by which the packaging state of chromatin, the histones in nucleosomes, and the corresponding DNA molecules are altered during processes such as replication and recombination of gene expression (3). Chromatin accessibility is one of the categories of chromatin remodeling and refers to the extent to which eukaryotic DNA can bind to other regulatory factors after binding to components such as nucleosomes or transcription factors (TFs). These properties of chromatin reflect relatively early alterations in chromatin dynamics in the face of various endogenous mutations and environmental stresses and play an important role in physiological and pathological processes (4, 5).

Digestive system cancers rank first in tumor-associated death and rank second in the new case chart after reproductive system cancers (6). Since epigenetics was introduced in the 4th edition of the WHO classification of digestive system tumors in 2010, we have gained a deeper understanding of the etiology and pathogenesis of digestive system tumors (7). However, not all tumors and phenotypes have been studied at the level of chromatin dynamics, and available studies do not investigate chromatin regulation at the genome-wide level.

Here, we provide a brief overview of chromatin structure, chromatin remodeling, and chromatin accessibility, the landmark studies pertaining to their roles in digestive system tumors, and we also summarize relevant clinical trials and posit new directions for future research and therapeutic approaches.

## Chromatin Disturbances and Regulatory Modifications in Digestive System Cancer

The concept “chromatin” was first coined by W. Flemming in 1880 (8). Chromatin is a moniliform complex composed of DNA, histones (H1, H2A, H2B, H3, and H4), non-histone proteins (enzymes that participate in DNA transcription and duplication), and a small amount of RNA forming in the nucleus during the interphase of the cell cycle (9). The structural monomers of chromatin, also termed the primary structure of chromatin, are nucleosomes. A nucleosome consists of an octamer of the four core histones encircled by 145~147 bp of DNA (10). Nucleosomes then coil, six per turn, and form the “solenoids” with an outer diameter of 30 nm, an inner diameter of 10 nm, and a pitch of 11 nm, which is called the secondary structure of chromatin (11). Subsequently, a cylindrical structure with a diameter of 0.4 μm will be formed by spiralization of the solenoids, which is named “supersolenoid,” the tertiary structure of chromatin (12). Finally, the supersolenoids fold and form the quaternary chromatin, namely, chromosome. Topologically associating domains (TADs) emerge as a fundamental structural unit in the spatial organization of the genome that is thought to guide regulatory elements to cognate promoters. Disruption of TADs by chromatin rearrangements, such as chromatin remodeling, and histone modifications can result in gene misexpression and pathogenesis (13).

**Figure 1:**
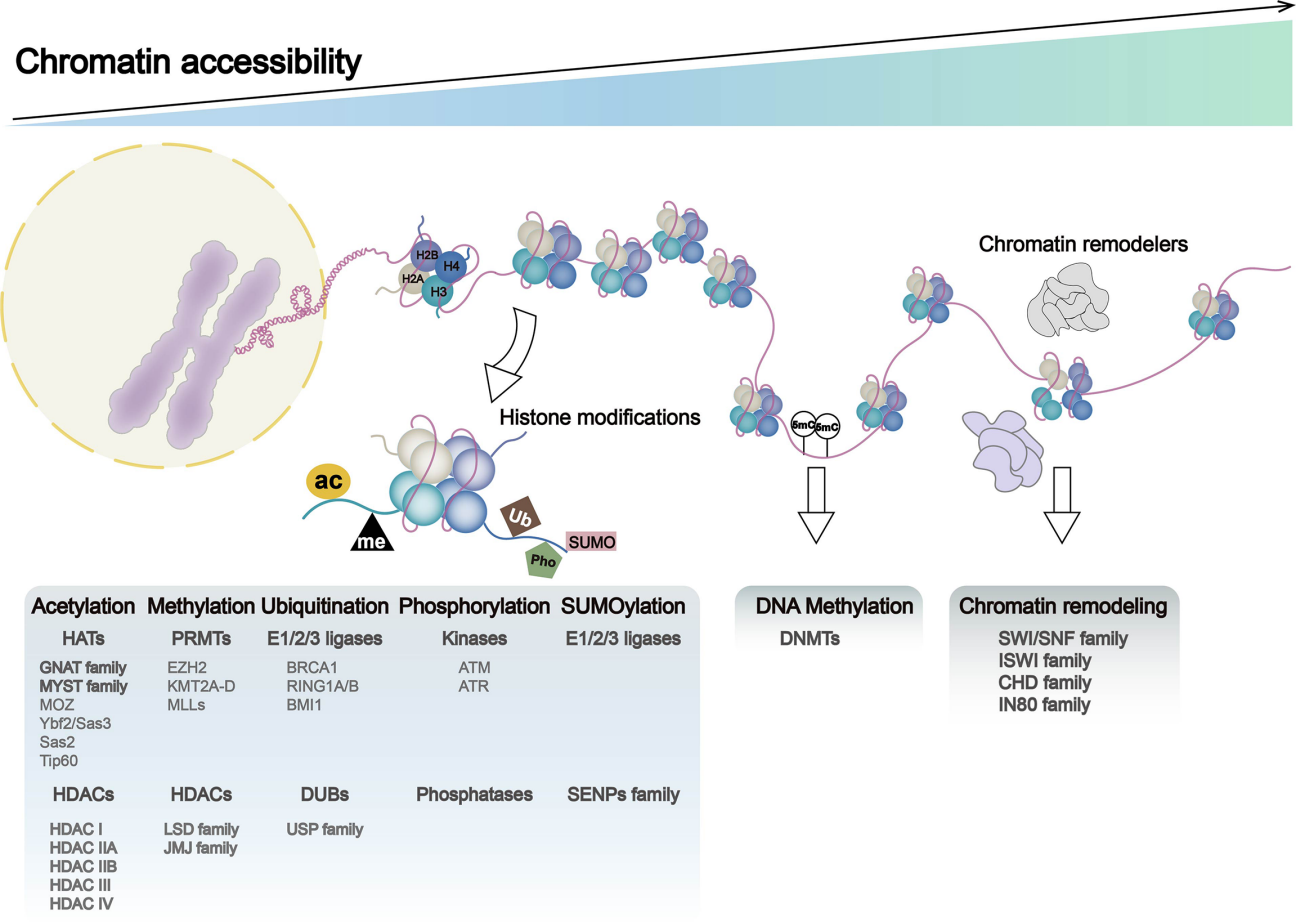
Chromatin dynamics in genome: chromatin accessibility, histone modification, DNA methylation, and chromatin remodeling. DNA entangles histones (H2A, H2B, H3, and H4) to form nucleosomes, the basic functional unit of chromatin. Nucleosome occupancy in the genome, histone modifications, DNA methylation, and chromatin remodelers leads to alternations in chromatin accessibility, which regulates processes such as gene transcription and translation. Histone modifications include histone methylation, acetylation, ubiquitination, phosphorylation, and SUMOylation, with histone-modifying enzymes and associated gene expression abnormalities playing a major role in these processes. Chromatin remodeling complexes include SWI/SNF, ISWI, CHD, and IN80.

**Figure 2:**
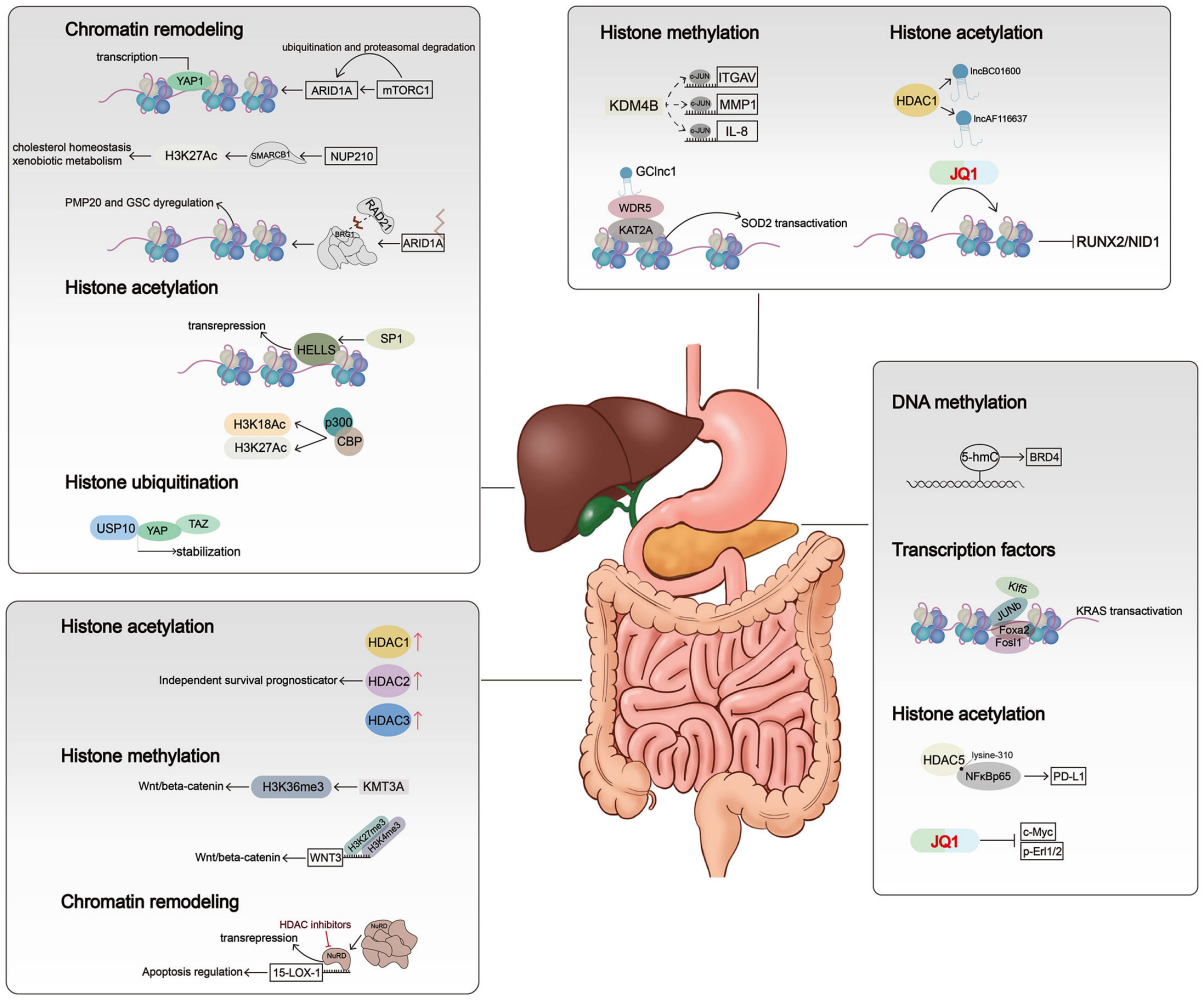
Mechanisms of chromatin alterations regulating digestive system tumors. Histone modifications, chromatin remodelers, and DNA methylation affect critical signal pathways not only by altering gene expression levels but also by regulating chromatin accessibility.

Chromatin remodeling and histone modifications may induce altered chromatin accessibility, and these three make major contributions to genome rearrangements. Chromatin accessibility was once termed as “a window into the genome,” which refers to other factors’ degree to physically rebind eukaryotic chromatinized DNA after histones and chromatin-binding factors bind to it (14). Dynamic change of chromatin accessibility constantly regulates DNA-based transactions including transcription, DNA replication, and repair. Factors such as nucleosome position and occupancy rate in the genome, chromatin remodeling complexes, histone modification, and DNA methylation are vital in determining and regulating the degree of chromatin accessibility. Histone modifiers, chromatin remodelers, and DNA modifiers dynamically regulate chromatin accessibility in different ways, such as ejecting nucleosomes and mutual charge repulsion. In this section, we summarized the effects of histone modifications, DNA modifications, and chromatin remodelers on chromatin accessibility (**Figure 1**) and their roles in the development of gastrointestinal tumors (**Figure 2**) (**Table 1**).

**Table 1:** Chromatin regulation and relative pathways in digestive system tumors.

Epigenomic modification	Cancer type	Element	Relative gene	Downstream effect	Reference
Histone acetylation	Hepatocellular carcinoma	P300/CBP	H3K27 H3K18	Chromatin accessibility	(15)
	Gastric cancer	HDAC1	lncBC01600 lncAF116637	/	(16)
	Gastric cancer	JQ1	RUNX2/NID1	Chromatin accessibility	(17)
	Pancreatic cancer	HDAC2	PDGFRa PDGFRb EGFR	TGF-β	(18)
	Pancreatic cancer	HDAC5	NF-κB p65	PD-L1	(19)
Histone methylation	Gastric cancer	GClnc1	SOD2 WDR5 KAT2A	Chromatin accessibility	(20)
	Hepatocellular carcinoma	NASP	P53 c-Myc	Chromatin accessibility	(21)
	Gastric cancer	KDM4B	IL-8 MMP1 ITGAV	/	(22)
	Colorectal cancer	KMT3A	WNT3	Wnt/β-catenin	(23, 24)
	Colorectal cancer	DPY30	ABHD5/YAP/c-Met	Chromatin accessibility	(25)
Histone ubiquitination	Cholangiocarcinoma	BAP1	/	Cell invasion and adhesion Cytoskeleton assembly-proteins	(26)
	Hepatocellular carcinoma	USP10	YAP/TAZ	/	(27)
SUMOylation	Pancreatic cancer	SAE2/UBA2, SAE1/UBE2I	MYC	MYC	(28)
DNA methylation	Colorectal cancer	DNMT	/	Wnt/β-catenin	(29)
5-Hydroxymethylcytosines	Pancreatic cancer	BRD4	/	/	(30)
SWI/SNF	Hepatocellular carcinoma	HELLS	CDH1	EMT	(31)
	Hepatocellular carcinoma	ARID1A	BRG1–RAD21	Chromatin accessibility	(32)
	Hepatocellular carcinoma	SMARCB1	NUP210	Chromatin accessibility	(33)
	Hepatocellular carcinoma	ARID1A	mTORC1	Chromatin accessibility	(34)
	Pancreatic cancer	ARID1A	ALDH1A1	KRAS	(35)
Regulation of transcription factors	Pancreatic cancer	KRAS mutation	Junb Fosl1 Klf5 Foxa2	Chromatin accessibility	(36)
	Pancreatic cancer	KRAS mutation	BRD4 IL33	Chromatin accessibility	(37)
	Intrahepatic cholangiocarcinoma	MFAP5	Notch1	Chromatin accessibility	(38)

### Histone Modification and Chromatin Accessibility in Digestive System Cancers

Histone tail modifications and the proteins that control them represent important components of chromatin regulation. Various types of chemical modification of histones such as histone acetylation, methylation, and ubiquitination give dynamic changes to nucleosome occupation and chromatin stages (39). Histone modifications exert their effect on the chromatin stage mainly through two mechanisms. First, the modifications directly influence the whole structure of chromatin, either short or long distances. Second, the modifications control the access of effector molecules. Since the discovery of highly transcriptional regions accompanied by hyperacetylated histones, over 150 different histone modification types have been identified, and their dysregulation can lead to inappropriate activation of oncogenes or, conversely, inactivation of tumor suppressors (40, 41).

#### Histone Acetylation

Histone acetylation, the first unveiled and most-studied histone modification type, was introduced in 1961, and the first histone acetyltransferase (HAT) and first histone deacetylase (HDAC) were discovered in 1996 (42). HATs (including P300/CBP, MYST family, and GNAT family) act on specific histone lysine residues in all four kinds of histones, thereby neutralizing the positive charge of lysine residues, weakening the charge-dependent association between histones and DNA or adjacent histones, and thus facilitating various factors’ contact to the loose region and making chromatin more accessible (43). Thus, histone acetylation is considered an active histone mark (44). The cyclic AMP response element-binding protein (CBP) often acts in conjugation with HATs P300 to form a CBP/P300 complex, which can further recruit other HATs like PCAF (P300/CBP-associated factor). Bi-allelic mutations of CBP and P300 have been observed in several cancers including colon cancer, breast cancer, and gastric cancer (45). The acetylation process can be reversed by HDACs, which tightly bind to negatively charged DNA and recover chromatin compaction. In humans, there are 18 HDACs belonging to four classes: the class I Rpd3-like proteins (HDAC1–3 and HDAC8), the class II Hda1-like proteins (HDAC4–7, HDAC9, and HDAC10), the class III Sir2-like proteins (SIRT1–7), and the class IV protein (HDAC11) (46). The class I, II, and IV HDACs are zinc-dependent, and the class III HDACs use NAD+ to generate nicotinamide and metabolite 20-*O*-acetyl-ADP-ribose during the process of deacetylation (47, 48). Dysregulation of class I and IV HDACs has been observed in gastric cancer, liver cancer, and colorectal cancer. For example, HDAC1 is involved in the promotion of gastric cancer cell proliferation, possibly by upregulating the expression of lncRNAs BC01600 and AF116637 in the tissues of patients with gastric cancer (16). In colorectal cancer, overexpression of HDAC1, HDAC2, and HDAC3 has been found, and HDAC2 expression was identified as an independent survival prognosticator (49). HDAC2 controlled the expression of pro-survival receptor tyrosine kinases connected to mesenchymal pancreatic ductal adenocarcinoma (PDAC), including PDGFRa, PDGFRb, and EGFR. The HDAC2-maintained program disrupted the tumor-suppressive arm of the TGF-β pathway, explaining impaired metastasis formation of HDAC2-deficient PDAC (18). HDAC5, a class IIa HDAC member, is downregulated in pancreatic cancer. HDAC5 regulates PD-L1 expression by directly interacting with NF-κB/p65 and reduces acetylation of p65 at lysine-310. Inhibition of HDAC5 sensitizes PDAC to immune checkpoint blockade (ICB) therapy (19). Several HDAC inhibitors (HDACis) have been approved by the US Food and Drug Administration (FDA) for cancer treatment or are currently being evaluated in clinical trials. Given that HDACi monotherapy has been largely ineffective in solid tumors such as pancreatic cancer and liver cancer, the evaluation of combination regimens is currently ongoing (50).

Triggering changes in chromatin accessibility is another important mechanism by which histone acetylation affects (positively or negatively) tumor progression. Histone acetylation reduces the positive charge of histones and disrupts electrostatic interactions between histones and DNA. This leads to a more accessible chromatin structure, thereby facilitating DNA access by molecules such as TFs or protein elements. Evidence for this can be found in loci within a hyper-acetylated and transcriptionally competent chromatin environment that shows higher DNase sensitivity and therefore are generally accessible (51). Gastric cancer-associated lncRNA1 (GClnc1) upregulates superoxide dismutase 2 mitochondrial (*SOD2*) transcription by acting as a scaffold to recruit the WDR5 and KAT2A complexes to the *SOD2* promoter, increasing levels of H3K4 trimethylation and H3K9 acetylation in the *SOD2* promoter region and leading to increased chromatin accessibility (20). P300/CBP mediates increased acetylation of H3K18 and H3K27 leading to hepatocellular carcinoma progression, and a novel P300 inhibitor, B029-2, exerts an antitumor effect by reducing amino acid metabolism and nucleotide synthase gene (including *PSPH*, *PSAT1*, *ALDH18A1*, *TALDO1*, *ATIC*, and *DTYMK*) promoter regions of H3K18Ac and H3K27Ac levels, leading to decreased chromatin accessibility and antitumor effects (15). The bromodomain and extraterminal domain (BET) family contains proteins, such as BRD3 and BRD4, that alter chromatin accessibility by recognizing acetylated histone lysine residues and accumulate on hyperacetylated chromatin regions that act as active promoters or enhancers and recruit TFs and multiprotein complexes to facilitate transcription of target genes. The small-molecule BET inhibitor JQ1 masks the bromodomain acetyl-lysine binding pockets and is highly specific for BET family proteins, particularly bromodomain 4 (BRD4)-containing proteins. In gastric cancer, JQ1 downregulates chromatin accessibility and inhibits the RUNX2/NID1 signaling pathway, thereby preventing gastric cancer progression (17). JQ1 is also widely used in other gastrointestinal tumors. In pancreatic cancer, JQ1 inhibits pancreatic cancer cell proliferation by reducing c-Myc and p-Erl1/2 protein levels (52). It has also been shown that gemcitabine and JQ1 act synergistically in pancreatic cancer through the LXR/RXR activation pathway (53).

#### Histone Methylation

Histone methylation is a covalent modification that occurs at the lysine (K) residues of histone H3 and H4 by adding methyl groups, which is one of the most important post-transcriptional modifications. The methylation is catalyzed by the histone methyltransferase (HMT), which uses *S*-adenosyl-l-methionine (SAM) as the substrate to transfer methyl groups onto the lysine residues of histones. The amine group of lysine residues may bind one (mono-), two (di-), or three (tri-) methyl moieties (54, 55). The known methylation loci on histones were H3K4, H3K9, H3K27, H3K36, H3K79, and H4K20. Among these methylation loci, H3K4, H3K36, and H3K79 were found in highly active transcription regions, whereas H3K9 and H4K20 are hallmarks of silent transcription or heterochromatin (54). Histone arginine methylation is found to mostly happen on H3R2, H3R8, H3R17, H3R26, and H4R3 (56). Methyltransferases have quite a specificity in recognizing residues and modification states due to the sharing catalytic core, the SET domain. The protein arginine *N*-methyltransferase (PRMT) family leads the process of histone arginine methylation, which is considered less specific than the lysine methyltransferases (57). By increasing the affinity of protein structural domains for histone tails, the stability of nucleosomes is increased, and heterochromatin formation is promoted (55). Histone demethylase (KDM) includes the LSD family and JMJ family. LSD1 with FAD as cofactor forms complexes with Co.REST, BHC80, and HDAC1/2, and other proteins play a biological role (58). JMJ family has a JmjC domain, with Fe(ii) and Ot monoketoglutarate as cofactors, which can demethylate multiple sites such as H3K4, H3K9, and H3K36 (59). Regarding gastrointestinal carcinogenesis, histone lysine demethylase 4B (KDM4B) physically interacts with c-Jun at the promoter loci of *IL-8*, *MMP1*, and *ITGAV* through its demethylation activity, and infection with *Helicobacter pylori* results in a significant increase in the occupancy of KDM4B and c-Jun, leading to a significant attenuation of H3K9me3 signaling (22). In addition, another study identified three H3K27me modifier genes (*EZH2*, *KDM6A*, and *KDM6B*) that are individually associated with GC susceptibility through a synergistic triad of actions (60). As reported, all inter-single-nucleotide polymorphism (inter-SNP) interactions among these three genes together form a synergistic triad epistasis network of ring-type topology. The *EZH2–KDM6B* interaction is significant, but *EZH2–KDM6A* and *KDM6B–KDM6A* interactions are merely marginal. In colorectal carcinogenesis, mutations in Wnt/β-catenin signaling mediators may be among the earliest events that initiate and drive tumor progression. In the absence of *KMT3A*, the activity of the Wnt/β-catenin signaling pathway is enhanced due to a marked reduction in H3K36me3, which drives colorectal carcinogenesis (23). Furthermore, another study showed the decrease in H3K27me3 and the increase in H3K4me3 in the *WNT3* promoter region, suggesting that histone methylation directly activates the Wnt/β-catenin signaling pathway and promotes CRC initiation (24).

There is no precise conclusion as to how histone methylation affects chromatin accessibility. Some histone methylation patterns (H3K4 and H3K79 methylation) seem to be necessary for the binding of TFs. Several studies have shown that histone methylation can affect the higher-order chromatin structure by recruiting chromatin remodeling complexes. For instance, BPTF, the component of the chromatin remodeler NURF, contains a PHD finger that recognizes H3K4me3 (61). DPF3, the component of the BAF complex, contains a double PHD finger that interacts with methylated histones (62). Nuclear autoantigenic sperm proteins (NASPs) are molecular chaperones of histones, and deletion of NASP leads to cell cycle accumulation at the S phase and failed replication (63). NASP deficiency induces histone pool disruption, mainly decreasing soluble H3, reducing H3K9me1 modification, and consequently causing chromatin to be more accessible, which helps prevent the development of hepatocellular carcinoma. With reduced cell proliferation due to NASP deletion, the expression levels of the pro-oncogenes *p53* and *c-Myc* were also decreased (21). In colorectal cancer, cancer stemness represents a major source of development and progression of CRC cells. The lipolytic factor *ABHD5* has been identified as an important tumor suppressor gene in CRC. Loss of *ABHD5* promotes c-Met activation to sustain CRC stemness in a non-canonical manner. Mechanistically, *ABHD5* interacts with the core subunit of the SET1A methyltransferase complex, DPY30. In the absence of *ABHD5*, DPY30 will translocate to the nucleus and support SET1A-mediated methylation of YAP and histone H3, which sequesters YAP in the nucleus and increases chromatin accessibility to promote *YAP*-induced transcription of c-Met (25).

#### Histone Phosphorylation

Histone phosphorylation, one of the most common post-translational modifications (PTMs), occurs at serine and tyrosine residues of histone proteins. Histone phosphorylation plays a similar role to histone acetylation in modulating nucleosome dynamics. Modified residues are imparted with a negative charge by phosphorylation, creating charge repulsion between histone and negatively charged DNA backbone, so that the association between DNA and histones can loosen and is less able to inhibit DNase I digestion (64, 65). In the human genome, histone H2A variant histone H2A.X is transformed into γH2A.X after phosphorylation at serine 139; this transformation is an essential part of the cellular response to DNA double-strand breaks (66). When phosphorylated by ATM or ATR kinases, γH2A.X recruits DNA repair-associated components to the double-strand break. It was also hypothesized that γH2A.X increases the level of chromatin accessibility to repair factors through charge repulsion (67). Histone phosphorylation can also alter the affinity of chromatin-binding proteins for their target molecules. For example, HP1 has a high affinity for H3K9me3, and when H3 serine 10 is phosphorylated, the binding of the HP1 chromosome group with H3K9me3 is inhibited (68–70).

#### Histone Ubiquitination

Histone ubiquitination includes monoubiquitination and poly-ubiquitination and results in a much larger covalent modification. The process of ubiquitination relies on three ubiquitin-activating enzymes. Ubiquitin-activating enzyme 1 (E1) first activates ubiquitin in an ATP-dependent manner and then binds to a cysteine residue of the ubiquitin-conjugating enzyme (E2) *via* a thioester bond. Finally, ubiquitin is transferred from the E2 enzyme to target lysine residues of specific substrate proteins by ubiquitin-protein isopeptide ligase (E3) (71). The process of ubiquitination can be reversed by deubiquitinating enzymes (DUBs). DUBs hydrolyze ester bonds, peptide bonds, or isopeptide bonds at the carboxyl terminus of ubiquitin, specifically separating ubiquitin from protein substrates and regulating the deubiquitination process. DUBs belong to the superfamily of proteases, including the ubiquitin deliberately modified enzyme family (USP). More than 90 DUBs have been identified, such as USP3, USP7, USP10, USP12, USP22, USP44, USP46, and USP49 (72). Immunohistochemical analyses revealed that aberrant histone ubiquitination patterns exist in many cancer types. Furthermore, DNA- and RNA-sequencing data show that genes encoding histone E3 ubiquitin ligases and DUBs are also frequently altered in cancers. RNF20 is the major H2B specific E3 ubiquitin ligase in mammalian cells. RNF20 represses gene expression by disrupting the interaction between TFIIS and the PAF1 elongation complex and inhibiting transcriptional elongation. Those effects are also dependent on the E3 ligase activity of RNF20 (73). In addition, RNF20-depleted cells show decreased expression of the *p53* and increased cell migration and tumorigenesis. USP22 is a ubiquitin hydrolase and catalyzes the removal of ubiquitin from monoubiquitinated histones H2A and H2B. In several studies, USP22 was found highly expressed in malignant tumor samples and associated with poor prognosis (74–76). Notably, USP22 has recently been found to function as a tumor suppressor in some tumors. For example, depletion of USP22 induced upregulation of secreted protein acidic and rich in cysteine (*SPARC*) by affecting H3K27ac and H2Bub1 occupancy on the *SPARC* gene in inflammation-associated colorectal cancer (77). In hepatocellular carcinoma, USP10 directly interacts with and stabilizes YAP/TAZ by reversing its proteolytic ubiquitination. This finding provides a rationale for potential therapeutic interventions in the treatment of patients with hepatocellular carcinoma harboring high levels of YAP/TAZ (27). BMI1 (also known as PCGF4), a member of PRC1 complex, can form homodimers and heterodimers with RING1 or PHC subunits9 that are critical for chromatin compaction. PTC596, a potent orally available BMI1 inhibitor, which can downregulate the anti-apoptosis factor MCL1, has progressed through phase I clinical trials for patients with advanced solid tumors (NCT02404480).

The effect of histone ubiquitination on chromatin accessibility is unclear, but some studies have shown that genes encoding ubiquitinases influence tumor progression by regulating chromatin accessibility. *BAP1* gene encodes a DUB and is identified as a tumor suppressor in many types of cancers including cholangiocarcinoma (78). *BAP1* targets multiple molecules and is involved in chromatin remodelers common with *PBRM1*, *IDH1*, *ARID1a*, and so on (78–80). With *BAP1* mutation, the ATAC-seq peaks were preferentially observed at TSS regions and the more accessible regions clustered in specific “hotspots” among the genome and a number of critical cell junction components; factors promoting cell invasion and adhesion and cytoskeleton assembly-proteins were noted to downregulate upon *BAP1* mutation in the global transcriptome. However, in *BAP1* mutation organoids, both decreases and increases in chromatin accessibility were observed in different genomic loci, which suggested that the function of *BAP1* might be divergent among various cell types (26). PRC1 complex contains a RING1 E3 ubiquitin ligase (RING1A/B), which catalyzes the monoubiquitylation of histone H2A (that is, H2AK119Ub) and PcG RING finger proteins (PCGF1–6).

#### SUMOylation

SUMOylation is an important PTM that fine-tunes virtually all cell functions and pathological processes. SUMOylation occurs through a cascade of enzymes similar to ubiquitination, but SUMOylation utilizes only a single conjugating enzyme, UBC9, and a certain number of ligases compared to complex ubiquitination. Humans express five SUMO paralogs, SUMO-1, -2, -3, -4, and -5 (81). SUMO molecules regulate the structure and function of substrate proteins by covalently binding to lysine residues of those with the participation of the E1-activating enzyme, E2-binding enzyme, and E3 ligase (81). SUMO-specific proteases (SENPs) regulate the SUMOylation state of substrate proteins together with SUMO molecules, by specifically deSUMOylating modification of substrate target proteins (82). SUMOylation is widely involved in DNA damage response (DDR) and regulates DNA damage sensing and repair protein, which is mainly found in chromatin and nuclear bodies (83). SUMOylation can block the binding sites of substrate proteins and interaction domains and can affect the function of proteins by blocking protein-interaction domains. SUMOylation can also produce new docking sites to facilitate the interaction with other proteins. MYC protein activates SUMO-activating enzyme subunit1 (SAE1) transcription by binding to canonical E-Box sequences located close to the SAE1 transcription start site. In pancreatic cancer, members of the SUMO pathway including *SAE2/UBA2*, *SAE1*, or *UBE2I*, have been found to synthesize lethal MYC interaction (28). TRIM family proteins have both SUMO E3 ligase and ubiquitin E3 ligase activities and are involved in multiple cellular processes including carcinogenesis. Overexpression of TRIM29 enhances cell proliferation and transforming activity and promotes tumor growth by reducing the acetylation of *p53* (84). Nuclear factor-κB (*NF-κB*) is an important TF for carcinogenesis in chronic inflammatory diseases and plays a key role in promoting inflammation-associated carcinoma in the gastrointestinal tract (85). TRIM40 promotes the neddylation of inhibitor of NF-κB kinase subunit γ and consequently causes the inhibition of *NF-κB* activity (86).

### DNA Methylation

DNA methylation and hydroxymethylation are important types of DNA modification in genome replication and transcription. DNA methylation plays a critical role in cell biology, including regulating gene expression, retro-element silencing, centromere stability and chromosome segregation in mitosis, X-chromosome inactivation, and monoallelic silencing of imprinted genes (87). In mammalian cells, DNA methylation is characterized by the addition of a methyl group at the carbon-5 position of cytosine base (5-methylcytosine (5-mC)) through the action of DNA methyltransferases (DNMTs) (87). 5-Hydroxymethylcytosine (5-hmC) is a further modified form of 5-mC, which is catalyzed by the Ten-Eleven Translocation (TET) protein family (88). DNA methylation mainly happens on “CpG islands” (clusters of CpG sites). CpG sites located within CpG islands are usually unmethylated in normal cells. They are activated in a transcriptionally permissive chromatin state that is characterized by combinations of post-translational histone modifications and special nucleosome organization (89). Unmethylated CpG sites within promoter CpG islands provide a binding platform for TFs to regulate gene expression (89), for example, specificity protein 1 (SP1), whose interactions with DNA are modulated by the presence or absence of DNA methylation at CpG islands (90). DNA methylation located in promoters is one of the most efficient patterns of gene transcription repression, which attributes to the function of remodeling chromatin. Until now, DNA methylation has been found to repress transcription in two ways. First, DNMTs can block the binding of transcriptional activators or coactivators with target sequences, thus directly inhibiting transcription initiation (91). Second, methyl-CpG-binding proteins (MeCPs) associated with chromatin remodelers can recognize DNA methylation sites and silence gene expression by recruiting co-repressors (92, 93). Distal regulatory regions such as tissue-specific enhancers are identified as CpG-poor and belong to lowly methylated regions (LMRs). It has been demonstrated that DNA methylation levels of enhancers are associated with gene activity at promoter–enhancer pairs, with a low level of 5-mC related to gene overexpression (94).

It is well known that global DNA methylation patterns are altered frequently in cancer development. Hypermethylation of CpG islands is common and mostly associated with the silencing of tumor suppressors, genes controlling cell growth, and downstream pathways. Numerous studies about locus-specific and genome-wide DNA methylation profiling have revealed multiple promoter-associated CpG islands that consistently undergo abnormal DNA hypermethylation in tumor cells (95). In addition, not only are single loci hypermethylated in cancer, but contiguous regions can become coordinately silenced and aberrantly hypermethylated. In colon cancer, CpG island Methylator Phenotypes (CIMPs) have been reported, enabling stratification of subtypes by a 5-mC signature (96). The expression of DNMT enzymes is also frequently disrupted in the tumor, which provides a feedback loop that drives alterations in DNA methylation patterns across the genome and has the potential to cause mutations in genomic sequence. Recently, DNMTs have been suggested as a potential epigenetic mechanism for maintaining cancer stem cells (CSCs). 5-Aza-2′-deoxycytidine (5-AzaDC), a novel DNMT inhibitor, was observed to significantly reduce the abundance of colorectal cancer CSCs and inhibit the growth of liver metastatic tumors by inhibiting the expression of active β-catenin and downregulating the Wnt signaling pathway (29).

During tumorigenesis, CpG-poor regions tend to undergo hypomethylation, resulting in the global decrease in DNA methylation characteristic of tumors. This phenomenon was first reported in colon adenocarcinoma and small cell lung cancer (97). DNA hypomethylation in cancer contributes to genomic instability and increased aneuploidy, both common features of cancer genomes. It is widely accepted that the global loss of DNA methylation in cancer cells is accompanied by widespread genomic instability. However, a causal relationship remains to be clearly shown. Extensive global hypomethylation regions are associated with global changes in chromatin organization and structural changes.

Alongside the global alternation of 5-mC, regulation in 5-hmC has been also observed in many cancer types. High-throughput sequencing of 5-hmC in the genome of mouse embryonic stem cells showed that 5-hmC was mainly enriched in the exons of totipotency genes and near the transcription start point, and this site was often accompanied by lysine trimethylation modification at position 4 of histone H3 (H3K4me3). 5-hmC content was positively correlated with chromatin state, and the phenomenon of decreased 5-mC content but increased 5-hmC occurred at multiple gene active transcription sites (98–100). Researchers conducted a comprehensive genome-wide analysis of 5-hmC in pancreatic cancer and found that 5-hmC could be detected in both PDAC and control non-neoplastic pancreatic epithelial cells, though its level was lower than that of 5-mC (101). Moreover, they also observed that variability of 5-hmC was mostly increased and ubiquitous in PDAC cell lines compared to healthy cells. According to the data acquired from ATAC-seq, 5-hmC regions (DHMRs) showed high chromatin accessibility, as expected. *BRD4* was found to acquire 5-hmC modification at regions overlapped with H3K4me1 peaks. Overexpression of *BRD4* is found to be tightly related to 5-hmC modification at the enhancer of the *BRD4* sequence. Bromodomain inhibitors including JQ1 can competitively bind to the acetyl-lysine recognition sites of BET family bromodomain, thereby displacing *BRD4* from nuclear chromatin and inhibiting cancer initiation. These kinds of molecular targeting inhibitors are already tested in early-phase clinical trials and are expected to become effective targeting drugs for cancer (30).

### Chromatin Remodelers

To achieve dynamic access to packaged DNA, cells have evolved a series of tailored regulation factors, named chromatin remodeling complex. The contribution of chromatin remodelers in regulating replication and transcription is obvious: i) specific remodelers can space nucleosomes correctly after replication to guarantee rational nucleosome position and properly arrange the whole genome. ii) Critical cis DNA elements are hidden among the densely packed nucleosomes, which lose the opportunity to interact with DNA-binding factors. Remodelers are able to slip the nucleosomes away and transiently expose the elements on the binding side. iii) The activities of DNA polymerases and RNA polymerases can be barriers to nucleosomes. Remodelers may help eject the nucleosomes or chaperone the histone octamers around the running polymerases (102). Because of this, chromatin remodeling complexes can be considered as important as other epigenetic mechanisms for oncogenesis. There are four different chromatin remodeler families that share a similar ATPase domain that has been identified: SWI/SNF family, ISWI family, CHD family, and INO80 family. The common properties of the four families are also described, including an affinity for the nucleosome, reorganization for covalent histone modifications, similar DNA-dependent ATPase domain, ATPase regulation domain, and chromatin or TF interaction domain. Apart from the common grounds, these four complexes are also special for their unique domains residing in catalytic ATPase and particular binding sites (103).

#### SWI/SNF Remodeler

SWI/SNF (switching defective/sucrose non-fermenting) family remodelers consist of 8 to 14 subunits. These family remodelers generally altered more than 20% of human malignancies (104). BAF and PBAF complex, whose specific ATPases were hBRM and BRG1, of the SWI/SNF family, are mainly included in human genome activity, and both contain a bromodomain (105). SWI/SNF plays a key role in chromatin remodeling and accessibility at promoters and enhancers by sliding and ejecting nucleosomes at multiple loci (106). Alternations in subunits of SWI/SNF complex and related genes play an important role in the development of digestive system tumors. For example, SNF2 is the most-studied example and interacts with various proteins including products of proto-oncogenes such as *p53*, *Rb*, and beta-catenin. HELicase, lymphoid-Specific (HELLS), also known as LSH, SMARCA6, or PASG, is a chromatin remodeling enzyme of the SNF2 family (107). Abnormal activity of TF SP1 in hepatocellular carcinoma leads to high expression of HELLS (31). At the epigenetic level, high HELLS expression increases nucleosome occupancy, decreases chromatin accessibility to enhancer regions, and inhibits the formation of nucleosome-free regions (NFRs) at TSSs. HELLS binds to the NFR of *CDH1*, which encodes E-cadherin and silences *CDH1* at the epigenetic level in hepatocellular carcinoma, thus contributing to EMT and cancer metastasis (31). SMARCB1, a subunit of the SWI/SNF complex, is significantly upregulated in hepatocellular carcinoma. SMARCB1 contributes to the stability of the BAF complex and its chromatin affinity. The putative tumor supporter, Nucleoporin210 (NUP210), is a critical coregulator of SMARCB1 chromatin remodeling activity, binds its enhancer, and alters H3K27Ac enrichment and downstream pathways, especially cholesterol homeostasis and xenobiotic metabolism (33).

The effect of the SWI/SNF family on chromatin accessibility has been most studied, including BRG1 (also known as SMARCA4), SNF5, BAF57, and BAF155 (108). *ARID1A* encodes a subunit of SWI/SNF, and its deletion in hepatocellular carcinoma induces conversion of the A/B region, remodeling of TADs, and a reduction in chromatin loops. RAD21 is a structural subunit of the chromatin structural element cohesin, and the ATPase BRG1 of the SWI/SNF complex can physically interact with RAD21. Lack of *ARID1A* markedly reduces BRG1–RAD21 coupling, leading to increased chromatin accessibility and promoting hepatocellular carcinoma metastasis (32). mTORC1 interacts with ARID1A protein in HCC and regulates ubiquitination and proteasomal degradation of ARID1A protein. The mTORC1–ARID1A axis promotes oncogenic chromatin remodeling, accessibility, and YAP-dependent transcription, thereby enhancing hepatocellular carcinoma cell growth *in vitro* and tumor development *in vivo* (34). Remarkably, *ARID1A* shows a high expression level in primary tumors but shows a decreasing trend in metastatic lesions, indicating that *ARID1A* may be an initiating factor in HCC and be lost in the later lesions (109). In pancreatic cancer, *ARID1A* deletion promotes pancreatic tumorigenesis by increasing chromatin accessibility to the enhancer region of aldehyde dehydrogenase 1 family member A1 (*ALDH1A1*), upregulating *ALDH1A1* expression, and attenuating *KRAS*-induced senescence (35).

#### ISWI Remodeler

ISWI (imitation switch) family remodelers include 2 to 4 subunits and are conserved from budding yeast to humans (110). This family is special for its attendant proteins and a characteristic set of domains located at the C-terminal of ISWI family ATPases. Until now, two primary ATPases, SNF2L (SNF2-”like”) and SNF2H (SNF2-”homolog”) complexes, were identified to be composed of three ISWI family complexes in mammalian cells, namely, NURF, CHRAC, and ACF complexes (111). Instead of leading to the disruption of nucleosomes, the ISWI family remodelers rebuild the gap between nucleosomes, thereby promoting chromatin assembly and lower chromatin accessibility and inhibiting transcriptional process (112, 113). SMARCA5, an ATPase of the ISWI class of chromatin remodelers, is dysfunctional in leukemia and breast, lung, and gastric cancers. Following conditional haplo- or duplex SMARCA5 deletion, cells undergo accelerated growth arrest, enter senescence, and show a progressive increase in susceptibility to genotoxic damage. These phenotypic features were interpreted as a specific remodeling of the chromatin structure and transcriptome of primary cells prior to the onset of immortalization (114).

#### CHD Remodeler

CHD (chromodomain, helicase, DNA binding) family includes two chromodomains tandemly arranged at the N-terminal of catalytic subunits in addition to ATPase (115). CHD family has been unveiled as a “double-edged sword” in transcription, some of which eject or slide nucleosomes away to promote transcription, while others show suppressive effects. This property of the CHD family may partly rely on chromodomain diversity (116). The suppressive role of the CHD family is partly contributed by the Mi-2/NuRD (nucleosome remodeling and deacetylase) complex, a member of the CHD family in high eukaryotes, and forms large protein complexes including HDAC subunits (117). 15-Lipoxygenase-1 (15-LOX-1) is transcriptionally silenced in colon cancer cells, and its reactivation restores apoptosis to cancer cells. NuRD contributes to 15-LOX-1 transcription suppression *via* recruitment to the promoter, while HDACis can dissociate NuRD from the promoter to activate 15-LOX-1 transcription (118).

#### INO80 Remodeler

INO80 (inositol requiring 80) family contains more than 10 subunits and was originally discovered as a protein necessary for transcriptional activation of the gene *ino1* (119). “Split” ATPase domain distinguishes the INO80 family from other chromatin remodeler complexes, with a long insertion present in the ATPase domain and binds with helicase-related Rvb1/2 proteins or another ARP protein. Thus, INO80 has unique significance in representing a new class of ATPases (120). INO80 family complex remodels nucleosome structure by exchanging classical and variant histones (121). However, the specific mechanisms of how INO80 affects epigenetic inheritance still need to be further explored.

### Transcription Factors

The fragments of accessible chromatin among the whole genome can be engaged with multiple binding factors, and the network between chromatin and TFs cooperatively controls the gene expression, playing an essential role in cancer development (4). With pancreatic cancer, normal pancreatic follicular cells are converted to duct-like cells in a process known as acinar-to-ductal metaplasia (ADM) (122–124). Meanwhile, a large number of pancreatic cancer precursor cells named pancreatic intraepithelial neoplasia (PanIN) are gradually generated in the pancreas of mice carrying *KRAS* mutation (125). Klf5 TF is highly expressed in human pancreatic cancer and is also expressed in normal pancreatic ductal cells and alveolar-to-ductal metaplasia (ADM). The KLF5-expressing ADM cells, called PDLP cells, have been shown to be a population of pancreatic cancer precursor cells that highly express a pro-oncogenic transcriptional regulatory network and have a strong differentiation capacity. Compared with normal pancreatic ductal cells, there are a large number of highly activated genes in PDLP, and the chromatin near these genes also becomes more accessible. The chromatin-accessible regions in PDLP cells are similar to those in PDAC cells. AP1, Ets, Fox, and Klf TF families are enriched in chromatin-accessible regions of PDLP, and the degree of chromatin accessibility is greatly downregulated after knockdown of Junb, Fosl1, and Klf5 (36). Pancreatitis associated with pancreatic tissue injury combined with *KRAS* mutation can also significantly accelerate the occurrence of early pancreatic cancer. Chromatin change associated with cancer initiation occurs within 48 h of pancreatic injury, indicating that chromatin remodeling changes occur at the initiation of pancreatic cancer (37). The cytokine interleukin-33 (IL-33) is rapidly activated in pancreatic tissue after injury (126). The presence of many IL-33-associated loci in the loose chromatin regions described above correlates with elevated *BRD4*-dependent IL-33 expression. Early in carcinogenesis, IL-33 links tissue damage with *KRAS* gene mutation-dependent epithelial plasticity to carcinogenesis (37). Microfibrillar-associated protein 5 (MFAP5) is an extracellular matrix (ECM) glycoprotein and a component of ECM microfibrils (38). Intrahepatic cholangiocarcinoma (ICC) patients with a higher level of MFAP5 are more likely with malignant progression and low survival rates. High expression of MFAP5 results in a more accessible chromatin landscape in specific regions, thereby promoting transcription of genes related to Notch1 pathways, subsequently accelerating the transition from G0/G1 phase to the S phase, and finally facilitating the aggressiveness of ICC.

### Mutations of Epigenetic-Related Genes in Digestive System Cancers

**Figure 3:**
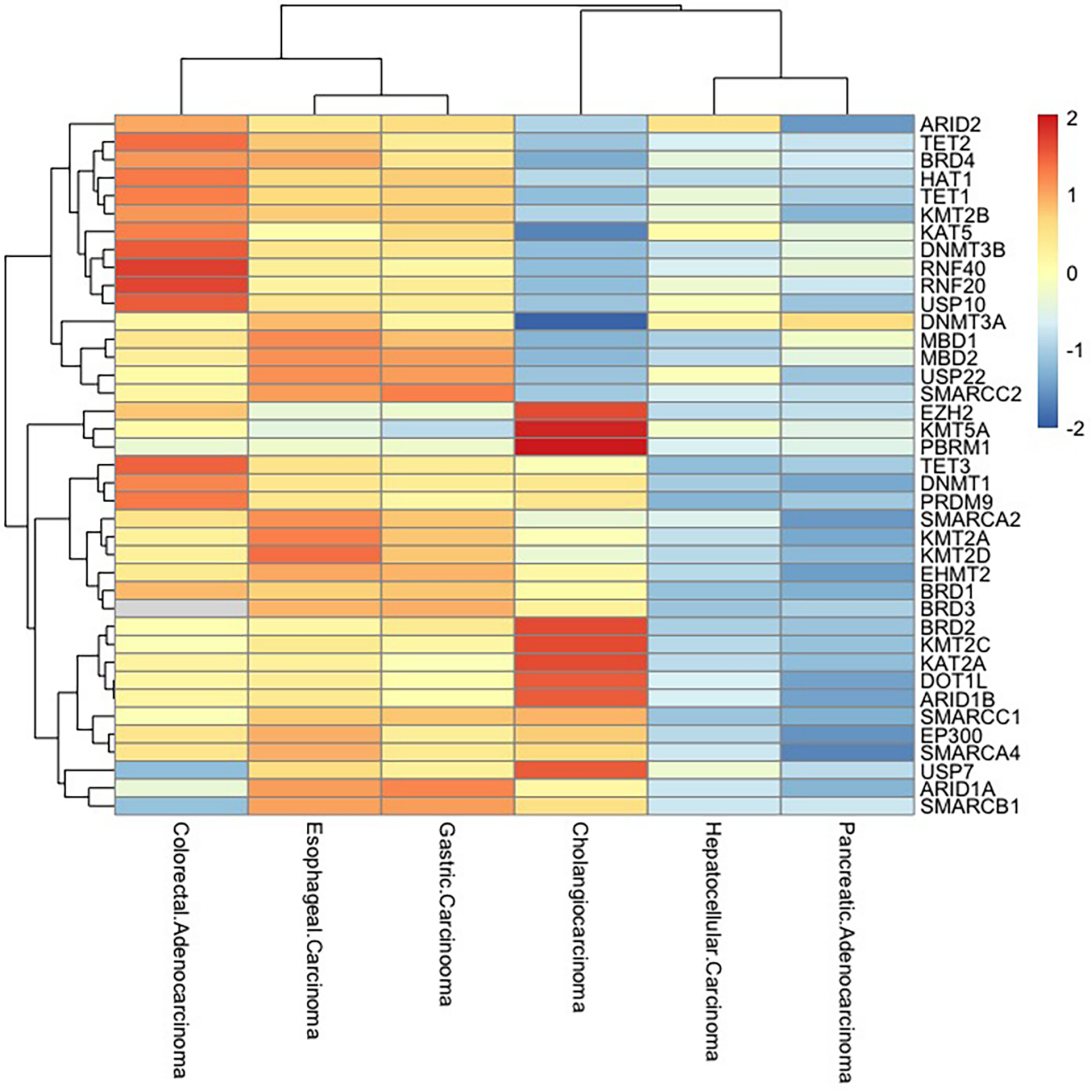
Epigenetic-related gene mutations in digestive system cancers. Frequency of mutations in epigenetically critical genes in digestive system tumors (esophageal carcinoma, gastric carcinoma, hepatocellular carcinoma, pancreatic adenocarcinoma, colorectal adenocarcinoma, and cholangiocarcinoma) is shown as a heatmap. The mutation rates of these genes are high in esophageal carcinoma, gastric carcinoma, and colorectal adenocarcinoma, while pancreatic adenocarcinoma and hepatocellular carcinoma have low mutation rates.

As epigenetic regulators, histone modification, DNA methylation, and chromatin remodelers are an important layer of transcriptional regulation with the particularity to affect gene expression. Over the years, due to a large number of recurrent mutations, hundreds of novel driver genes have been characterized in cancers. However, it seems not well-documented to consider cancer only as the end product of accumulated somatic mutations. There exist few cancers with a limited number of somatic mutations such as thyroid cancer and marker cell carcinoma. Despite epigenetic-related genes being far less in numbers than the genes directly linked to cancer, the global impact on the genome cannot be ignored. Herein, we summarized several critical gene mutations associated with DNA methylation, histone modification, and chromatin remodeler SWI/SNF complexes in digestive system cancers (**Figure 3**).

DNMT enzymes, mainly DNMT1, DNMT3A, and DNMT3B, catalyze/regulate DNA methylation. DNMT1 maintains the methylation status of newly replicated DNA strands, while DNMT3A and DNMT3B are responsible for *de novo* DNA methylation. A meta-analysis in gastric cancer suggested that rs16999593 in *DNMT1* and rs1550117 in *DNMT3A* could contribute to GC risk and that rs1569686 in *DNMT3B* might be a protective factor (127). DNA methylation is not limited to the effect of DNMTs. The TET family (including TET1, TET2, and TET3) catalyzes the transformation from 5-mC to 5-hmC. Missense and truncating mutations in *TET* genes have been observed in almost all tumor types with relatively low frequency (0.1%–10% of cases). In colorectal cancer, up to 20% of patients were found to carry mutations in one or more of the *TET* genes (http://www.cBioPortal.org). However, it seems like mutation types of *TET* genes in solid tumors are often missense mutations with no significance.

As a complicated and far-reaching epigenetic entity, the impacts of mutations in histone-modifying enzyme-associated genes on tumors remain in the research focus. There are numerous reports showing the involvement of mutations in genes encoding HATs (*EP300*, *P300*, *CBP*, *MOZ*, etc.) in many cancers. The EP300 protein is a HAT that regulates transcription and chromatin dynamics. Six mutations of *EP300* gene were analyzed in 193 epithelial cancers (128). Of the six mutations, two were in primary tumors (a colorectal cancer and a breast cancer) and four were found in cancer cell lines (colorectal, breast, and pancreatic). In addition, missense alterations were found in primary colorectal cancer and two cancer cell lines (breast and pancreatic). These data show that *EP300* is mutated in epithelial cancers and behaves as a tumor-suppressor gene. *UTX* (also known as *KDM6A*) as a highly mutated gene encoding histone H3K27 demethylase has been reported in several cancer cell lines including colorectal adenocarcinoma and pancreatic adenocarcinoma (129).

Several members/subunits from chromatin remodeling families, such as *hSNF5/INI1*, *ARID1A*, and *MTA1*, are known to be mutated in human cancers. In the cancer spectrum, SWI/SNF complex has gained particular attention, as they are mutated in nearly 20% of human cancers (130). The frequency of *ARID1A* mutation is 17% in gastric cancer patients and 12%–13% in colorectal cancer patients. The mutations of *AIRD1A* are significantly associated with microsatellite instability (MSI) and Epstein–Barr virus (EBV) infection and also poorly differentiated grade and advanced tumor depth (131).

## Developing Technologies for Measuring Chromatin Dynamics

As mentioned earlier, eukaryotic genomes are hierarchically packaged into chromatin, and various forms of packaging play different roles in gene expression and regulation. The shortcut to comprehending the epigenetic information encoded in the chromatin mainly comes from high-throughput, genome-wide methods, which focus on chromatin accessibility, nucleosome position, and TF occupancy. In this section, we summarized four existing assays for measuring the chromatin stage and their principles (**Table 2**).

**Table 2:** High-throughput methods for chromatin detection.

Methods	Core elements	Target region	Critical experimental steps	Reference
ChIP-seq	Immunoprecipitation	Whole genome Specified region	10^5^~10^7^ cells Crosslinking Sonication Immunoprecipitation DNA purification Library and sequencing	(132–134)
MNase-seq	Micrococcal nuclease	Nucleosome occupancy	Crosslink with formaldehyde MNase extracts nucleosomes High-throughput sequence	(135–142)
DNase-seq	Endonuclease DNase1	Chromatin opening region Nucleosome occupancy TF occupancy	Crawford Stamatoyannopoulos	(143–150)
FAIRE-seq	Formaldehyde	Chromatin opening region	Crosslink with formaldehyde Shearing chromatins with sonication phenol Chloroform extraction DNA detection PCR NGS	(14, 151–155)
ATAC-seq	Tn5 transposase	Chromatin opening region Nucleosome occupancy DNA binding protein	500–50,000 cells Tn5 as adaptors High-throughput DNA sequencing PCR NGS	(156–159)

ChIP-seq, chromatin immunoprecipitation sequencing; MNase-seq, micrococcal nuclease sequencing; DNase-seq, deoxyribonuclease sequencing; FAIRE-seq, Formaldehyde-Assisted Isolation of Regulatory Elements sequencing; ATAC-seq, assay for transposase-accessible chromatin using sequencing; NGS, next-generation sequencing.

Chromatin immunoprecipitation (ChIP) is the first technique to be applied to large-scale epigenetic mapping, followed closely by ChIP-chip to enable genome-wide detection of DNA–protein interactions (132). ChIP-chip is based on microarray hybridization. However, this method is not widely used due to its low resolution, ambiguous surface introduced by probe design, and signal bias. With higher resolution, less noise, and greater coverage, ChIP sequencing (ChIP-seq) is gradually becoming one of the indispensable tools for epigenetics as second-generation sequencing becomes popular (133). Based on ChIP-seq, the development of single-cell ChIP-seq (scDrop-ChIP, sc-itChIP-seq, etc.) has helped to study the genetic diversity of heterogeneous cell populations and understand the evolution of tumor populations, allowing the clustering of cell populations based on the diversity of chromatin landscapes and the identification of chromatin features specific to each cell population. The disadvantage of single-cell ChIP-seq is that thousands of cells are required to obtain good clustering results (134). ChIP-seq and scChIP-seq are now widely applied in research related to tumors. The molecular dependencies of pancreatic cancer were mapped through ChIP-seq, RNA-seq, and genome-wide CRISPR analysis and revealed an unexpected utilization of immunoregulatory signals by pancreatic cancer epithelial cells (160). In a 2021 study, ChIP-seq was used to profile active enhancers at the genome-wide level in colorectal cancer patient tissues. As a result, 5,590 gain and 1,100 lost variant enhancer loci, and 334 gain and 121 lost variant super enhancer loci were identified (161). RNA-seq, MBD-seq, and H3K27ac ChIP-seq on gastric tissues and cell lines were performed, and 257,651 significant differentially methylated regions were identified in gastric cancer, which provide insight for understanding methylation changes at distal regulatory regions and reveal novel epigenetic targets in gastric cancer (162).

As our understanding of the structure and dynamics of chromatin has improved, techniques for detecting chromatin accessibility have also made great strides. MNase (micrococcal nuclease), an endo- and exo-nuclease, could preferentially digest naked DNA between nucleosomes, releasing nucleosomes from chromatin and retaining the DNA fragments that are protected by nucleosomes (135). Early in 1970, MNase digestion has been applied to detect chromatin structure in low-throughput sequence and later applied in tiled microarrays (136–138). Nowadays, MNase mainly is used together with next-generation sequencing (NGS) to qualitatively and quantitatively assess nucleosome messages in the whole genome (139). MNase-seq combined with ChIP-seq can probe regulatory factors or histone-tail modification relative to nucleosomes (140). At the single-cell level, scMNase-seq reproducibly detects an average of ∼3, 0.9, and 700,000 unique fragments per cell type. The location of genome-wide nucleosomes in single cells is precisely defined, and subnucleosome-sized DNA fragments provide information on chromatin accessibility (141). However, MNase-seq has a lethal weakness, namely, sequence bias. It is easier and faster for MNase to cleave upstream of A or T, nearly about 30 times faster than it does on 5′ of G or C. Due to this bias in digesting level, careful and repeated enzymatic titrations must be supplied to improve the accuracy and credibility of MNase-seq (142).

Highly active regions of genomes commonly have an altering chromatin structure, thereby generating DNase hypersensitive sites (DHSs), which are chromatin accessible and can be cut by DNase1 (143). In the earlier DNase digestion assay, identification of DHSs relies on Southern blotting, and the detection regions on the genome were limited to a narrow range (144). Further improvement attempts to combine low-throughput sequence, real-time PCR, and hybridization to tiled microarrays. However, the efficiency and accuracy still remain unsatisfactory (145–147). DNase-seq ultimately became popular until the advent of NGS, which allows identifying DHSs among the whole genome specifically and sensitively (148). DNase-seq not only is able to unveil chromatin accessibility among distinctive cell lines but also has the ability to show the single nucleosome position (149, 150). Additionally, DNase-seq footprints can reveal that TFs occupy chromatin qualitatively and quantitatively (163). Single-cell DNase sequencing (scDNase-seq) detects genome-wide DHSs starting from <1,000 cells of single or primary cell origin, and about 50% of bulky DHS promoter sites can be detected (164). However, several studies have demonstrated that DNase1 introduced cleavage bias. Furthermore, TFs bind to DNA transiently in living cells and are not shown in DNase-seq footprints (165).

FAIRE was first reported by Nagy and Lieb in 2003 (151) and then formally named in 2007 (166). In FAIRE-seq, chromatins are crosslinked with formaldehyde first in order to catch *in vivo* protein–DNA binding and then shearing chromatins with sonication, followed by phenol–chloroform extraction and detection of DNA within the aqueous phase. The regions where nucleosomes are depleted will be released into the aqueous phase of the solution, and subsequently, the chromatin-accessible subgroups of fragments can be detected by real-time PCR, tiling DNA microarrays or paired-end/single-end NGS (151, 152). The advantage of FAIRE-seq is that it directly enriches areas of active chromatin while nucleosome-depleted regions are not degraded (153, 154). Furthermore, the sequence-specific bias in MNase and DNase is overcome in FAIRE-seq (155), although the limitations of FAIRE-seq cannot be ignored, including its lower signal-to-noise rate compared with other assays and difficulty in data computation due to this high background (14).

ATAC-seq was first thoroughly described as “fast and sensitive epigenomic profiling of opening chromatin” by Jason D. Buenrestro et al. in 2013. In ATAC-seq, information such as nucleosome package and position, and DNA binding sites can be read (156). Usage of Tn5 transposase is considered the core driver in creating this technique (157, 158). In ATAC-seq, the accessible regions of chromatin are more likely for Tn5 transposase to integrate its adaptor into and generate highly intensive peaks due to steric hindrance. In contrast, the regions of lower chromatin accessibility seem to set a barrier to such transposition (156). In 2015, single-cell ATAC-seq (scATAC-seq) was developed to detect transposase-accessible chromatin by using sequencing integrated into programmable microfluidic platforms (ATAC-seq), dissecting single-cell epigenomic heterogeneity, and linking cis and trans effectors to variability in the accessibility profile of individual epigenomes (159).

Currently, ATAC-seq is the most commonly used method to detect chromatin accessibility. For instance, ATAC-seq was used to investigate epigenetic elements responsible for the differential response to anti-PD-1 therapy by quantitatively assessing the genome-wide chromatin accessibility of circulating CD8+ T cells in patients’ peripheral blood. In this study, unique accessible regions of chromatin were identified to distinguish anti-PD-1 therapy responders from non-responders (167). Notably, ATAC-seq has been shown to have the potential to predict tumor prognosis. By ATAC-seq analyses of EpCAM+ PDAC epithelial cells sorted from 54 freshly resected human tumors, researchers found 1,092 chromatin loci displaying differential accessibility between patients with disease-free survival (DFS) < 1 year and patients with DFS > 1 year (168).

## Therapy Targeting

Over the past several decades, research on chromatin dynamics and its relationship with disease, particularly cancer, has provided us with strong evidence of its potential for cancer therapy. Dynamic change in genomic architecture caused by intricate cross-linking of elements of chromatin almost controls the function of every cell. As described before, the chromatin stage can be regulated on several levels such as DNA sequence and histone modification. The regulating patterns include chromatin remodeling complexes, methylation, and acetylation. Undergoing various types of modification on different levels, the accessibility of chromatin to regulatory elements such as TFs and modifying enzymes will be altered. Subsequently, the global genome landscape also is changed and affects the expression of the downstream gene. A series of actions cause positive or negative influences on the process of the cell cycle. Research concentrating on chromatin targeting therapy is ongoing and has gained rapid development in several hotspots such as HDACs, PRC2, and *EZH2* (169).

Drugs targeting chromatin remodeling complexes and histone modifications are actively being tested in clinical trials and approved by the US FDA (**Table 3**) (170, 171), such as histone deacetylation inhibitors, histone demethylation inhibitors, and drugs targeting the SWI/SNF chromatin remodeling complex. Regimens of the above drugs alone or combined with conventional chemotherapeutic agents have been addressed in several clinical trials.

**Table 3:** Clinical trials targeting epigenetic modifiers in digestive system cancers.

Target	Drug	Tumor type	Strategy	Phase	Status	NCT number
Histone methylation	Guadecitabine (inhibitor of DNA methyltransferase)	Hepatocellular Carcinoma	Guadecitabine	I/II	Completed	NCT01752933
		Colorectal cancer	Guadecitabine	I/II	Completed	NCT01896856
	Tazemetostat (Target EZH2)	Solid/advanced solid tumor	Tazemetostat	II	Recruiting	NCT05023655
			Itraconazole Rifampin Tazemetostat	I	Active, not recruiting	NCT04537715
			Tazemetostat	I	Recruiting	NCT04241835
			Tazemetostat	II	Active, not recruiting	NCT03213665
			Tazemetostat Durvalumab	II	Recruiting	NCT04705818
Histone acetylation	Vorinostat (inhibitor of HDAC1, HDAC2, HDAC3, HDAC6)	Pancreatic cancer	Vorinostat+capecitabine +radiotherapy	I	Completed	NCT00983268
		Gastrointestinal tumors	Vorinostat+pelvic radiation	I	Completed	NCT00455351
			Vorinostat+5-FU+irinotecan hydrochloride+leucovorin calcium	I	Completed	NCT00537121
		Gastric cancer	Vorinostat+capecitabine+cisplatin	I/II	Completed	NCT01045538
		Hepatocellular Carcinoma	Vorinostat+sorafenib tosylate	I	Completed	NCT01075113
	Domatinostat (inhibitor of class I HDACs)	Gastrointestinal tumors	Domatinostat	II	Unknown	NCT03812796
	Resminostat (Inhibitor of class I HDACs)	Cholangiocarcinoma	Reminostat+FOLFIRI	I/II	Completed	NCT01277406
		Gastrointestinal tumors	Reminostat+sorafenib	I/II	Completed	NCT00943449 NCT02400788
			Reminostat	II	Completed	NCT00098527
		Pancreatic cancer	Romidepin+azacitidine+nab-paclitaxel+gemcitabine Reminostat+nab-paclitaxel+gemcitabine	I/II	Recruiting	NCT04257448
			Reminostat+S-1	I	Completed	JapicCTI152,864
		Gastric cancer	Reminostat	II	Completed	NCT00077337
			Reminostat+FOLFIRI	I/II	Completed	NCT01277406
	Depsipeptide (inhibitor of class I HDACs)	Pancreatic cancer	Depsipeptide+gemcitabine	I/II	Completed	NCT00379639
Chromatin remodelers	Palbociclib (CDK4/6 inhibitor)	Pancreatic cancer	Palbociclib+ulixertinib	I	Active, not recruiting	NCT03454035
			Palbociclib	II	Completed	NCT02806648
			Palbociclib+ binimetinib	Early I	Recruiting	NCT04870034
		Gastrointestinal tumors	Palbociclib	II	Completed	NCT01907607
		Hepatocellular carcinoma	Palbociclib	II	Active, not recruiting	NCT01356628
		Colorectal cancer	Palbociclib+cetuximab	I	Active, not recruiting	NCT03454035
			Palbociclib+binimetinib	II	Active, not recruiting	NCT03981614
			Palbociclib+binimetinib	Early I	Recruiting	NCT04870034
			Palbociclib+ Cetuximab +Encorafenib+ERAS-007	I/II	Recruiting	NCT05039177
	Rucaparib (PARP inhibitor)	Pancreatic cancer	Rucaparib	II	Active, not recruiting	NCT03140670
	Dasatinib (targeting S100)	Pancreatic cancer	Dasatinib+placebo	II	Completed	NCT01395017
			Dasatinib+mFOLFOX6	II	Completed	NCT01652976
		Gastrointestinal tumors	Dasatinib	II	Completed	NCT00568750
	Bortezomib	Hepatocellular Carcinoma	Bortezomib+doxorubicin	II	Completed	NCT00083226
	Olaparib (PARP inhibitor)	Pancreatic cancer	Bortezomib+doxorubicin	II	Completed	NCT00083226

HDACis have been indicated as potent inducers of differentiation, growth arrest, and apoptosis induction. Vorinostat is a broad-based inhibitor of HDAC activity, inhibiting class I HDACs (HDAC1, HDAC2, HDAC3, HDAC8) and class II HDACs (HDAC6 and HDAC10, and HDAC11). Several clinical trials have been conducted to validate the use of vorinostat in combination with other chemotherapeutic agents (e.g., capecitabine and 5-FU) in colorectal cancer, pancreatic cancer, and other gastrointestinal tumors. Patients with gastrointestinal tumors (NCT00455351) showed better tolerability and stability when treated with vorinostat alone with a reduced dose (vorinostat 300 mg bid for 3 consecutive days followed by 4 days of rest) or combined with radiotherapy (172, 173). Pancreatic cancer patients showed good tolerance (NCT00983268) to the combination of vorinostat and capecitabine with radiation (174). Combinations of vorinostat with capecitabine, cisplatin, 5-FU, leucovorin, sorafenib tosylate, and other drugs have also been actively tried in several clinical trials on gastric cancer, colorectal cancer, and liver cancer. Resminostat is a new oral pan-HDACi that specifically targets HDAC1, HDAC2, and HDAC3. The effectiveness of resminostat in combination with several drugs such as sorafenib, cisplatin, and doxorubicin has been demonstrated. Resminostat combined with S-1 or FOLFIRI chemotherapy regimens has also been applied in patients with pancreatic cancer and colorectal carcinoma and demonstrated promising efficacy. However, according to the clinical trials mentioned above, the side effects of these drugs are not negligible, including diarrhea, anorexia, fatigue, and rash. Better regimens and dose assessments are yet to be proven.

Clinical trials targeting histone methylation modifiers have focused on the effects on hematological malignancies such as stomatous lymphoma and non-Hodgkin’s lymphoma. As for digestive system cancer, guadecitabine alone has been examined closely in phase I/II clinical trials of colorectal cancer and hepatocellular carcinoma (NCT01896856, NCT01752933). Guadecitabine was administered at two doses in patients with advanced hepatocellular carcinoma who had failed sorafenib treatment (NCT01752933). The median survival of included patients was 294 and 245 days, and the most serious adverse reaction was hematopoietic system dysfunction. Future research should pay more attention to these aspects in order to identify new treatment options for cancers of the digestive tract.

SWI/SNF has the broadest function of the four chromatin remodeling complexes, and drugs targeting this complex have been involved in several clinical trials, such as palbociclib, olaparib, rucaparib, bortezomib, and abemaciclib. Pancreatic neuroendocrine cancer patients with palbociclib alone had an overall survival of 33 months (NCT02806648). In patients with PDAC treated with abemaciclib+LY3023414+gemcitabine+capecitabine in different combinations, the overall survival was only about 6–10 months (NCT02981342).Conclusion and perspective

The regulation of chromatin dynamics by transcriptional elements and related complexes affects various pathways of digestive system tumor development, metastasis, and drug resistance and provides complex and precise control of various biological behaviors including cell cycle, metabolic program, and tumor microenvironment. The individual heterogeneity of tumors poses a very serious challenge for clinical treatment, and chromatin, which integrates genetic and epigenetic information, is a promising avenue to realize personalized treatment.

In the past decade or so, tremendous progress has been made in the field of chromatin regulation and cancer mechanisms, owing to in-depth investigations of chromatin regulatory factors, how these regulatory elements act on tumors, and attempts of targeting drugs in clinical therapy. In parallel, the invention of sequencing technologies such as ATAC-seq has further advanced our understanding of chromatin regulatory features, histone modifications, etc.

The current exploration of chromatin dynamics is primarily restricted to the regulation of extra-chromatin factors. It is worthwhile to consider whether chromatin already has potential accessible features in the early stage of formation. What is more, chromatin modulation-based tumor treatment strategies are rarely used in clinical training. First, there are still many limitations in regulating gene networks at the chromatin level, for example, the escape mechanisms and complexity of tumor signaling pathways under various stress stimuli, as well as diversities in the expression of a gene in a large patient population and the individual heterogeneity of downstream signaling pathways in each patient. Second, existing techniques for determining chromatin status still have many drawbacks and limitations, and there are no methods that can present the complete and dynamic genomic status of tumor patients and intervene. Application to individualized tumor treatment still requires much exploration and a long-term course of clinical trials. With the development of chromatin characterization and application, individualized tumor therapy is becoming unveiled.

## Author Contributions

ZL and BZ contributed equally to this review. WW, BZ, and ZL raised the concept. ZL conducted the literature review and wrote the first draft. BZ and CQ drew the figures. TL and YW organized the tables. All authors revised and approved the final manuscript.

## Funding

The research was funded by the National Natural Science Foundation of China, grant number (Nos. 81773215 and 82173074) and CAMS Innovation Fund for Medical Science (CIFMS), grant number (2021-12M-1-002).

## Conflict of Interest

The authors declare that the research was conducted in the absence of any commercial or financial relationships that could be construed as a potential conflict of interest.

## Publisher’s Note

All claims expressed in this article are solely those of the authors and do not necessarily represent those of their affiliated organizations, or those of the publisher, the editors and the reviewers. Any product that may be evaluated in this article, or claim that may be made by its manufacturer, is not guaranteed or endorsed by the publisher.

## Glossary

**Table:**

TADs	topologically associating domains
HAT	histone acetyltransferase
HDAC	histone deacetylase
HDACi	HDAC inhibitor
PDAC	pancreatic ductal adenocarcinoma
CBP	cyclic AMP response element-binding protein
ICB	immune checkpoint blockade
GClnc1	gastric cancer-associated lncRNA1
SOD2	superoxide dismutase 2 mitochondrial
BET	bromodomain and extraterminal domain
BRD4	bromodomain 4
HMT	histone methyltransferase
SAM	*S*-adenosy-{{sc}}l{{/sc}}-methionine
PRMT	protein arginine *N*-methyltransferase
KDM	histone demethylase
NASPs	nuclear autoantigenic sperm proteins
PTMs	post-translational modifications
DUBs	deubiquitinases
E1	ubiquitin-activating enzyme 1
E2	ubiquitin-conjugating enzyme
E3	ubiquitin-protein isopeptide ligase
USP	ubiquitin deliberately modified enzyme family
SENPs	SUMO-specific proteases
DDR	DNA damage response
NF-κB	nuclear factor-κB
5-mC	5-methylcytosine
DNMTs	DNA methyltransferases
MeCPs	methyl-CpG-binding proteins
5-hmC	5-hydroxymethylcytosine
TET	Ten-Eleven Translocation
SWI/SNF family	switching defective/sucrose non-fermenting family
ISWI family	imitation switch family
SNF2L	SNF2-”like
SNF2H	SNF2-”homologue”
CHD family	chromodomain, helicase, DNA binding family
NuRD	nucleosome remodeling and deacetylase
INO80 family	inositol requiring 80 family
HELLS	HELicase
NFRs	nucleosome-free regions
ADM	acinar-to-ductal metaplasia
PanIN	pancreatic intraepithelial neoplasia
ICC	intrahepatic cholangiocarcinoma
MFAP5	microfibrillar-associated protein 5
ECM	extracellular matrix
LTR	long terminal repeat
GC	gastric cancer
HCC	hepatocellular cancer
ChIP	chromatin immunoprecipitation
ChIP-seq	chromatin immunoprecipitation sequencing
ATAC-seq	assay for transposase-accessible chromatin using sequencing
NGS	next-generation sequencing
scATAC-seq	single-cell ATAC-seq
MNase	micrococcal nuclease
MNase-seq	micrococcal nuclease sequencing
DNase-seq	deoxyribonuclease sequencing
DHSs	DNase hypersensitive sites
scDNase-seq	single-cell DNase sequencing
FAIRE-seq	Formaldehyde-Assisted Isolation of Regulatory Elements sequencing
